# Micellar catalysis of the Suzuki Miyaura reaction using biogenic Pd nanoparticles from *Desulfovibrio alaskensis*†

**DOI:** 10.1039/d1gc02392f

**Published:** 2021-10-11

**Authors:** Yuta Era, Jonathan A. Dennis, Stephen Wallace, Louise E. Horsfall

**Affiliations:** Institute of Quantitative Biology, Biochemistry and Biotechnology, School of Biological Sciences, University of Edinburgh Roger Land Building Alexander Crum Brown Road King's Buildings Edinburgh EH9 3FF UK louise.horsfall@ed.ac.uk stephen.wallace@ed.ac.uk; School of Chemistry, University of Edinburgh Joseph Black Building David Brewster Road King's Buildings Edinburgh EH9 3F UK

## Abstract

Microorganisms produce metal nanoparticles (MNPs) upon exposure to toxic metal ions. However, the catalytic activity of biosynthesised MNPs remains underexplored, despite the potential of these biological processes to be used for the sustainable recovery of critical metals, including palladium. Herein we report that biogenic palladium nanoparticles generated by the sulfate-reducing bacterium *Desulfovibrio alaskensis* G20 catalyse the ligand-free Suzuki Miyaura reaction of abiotic substrates. The reaction is highly efficient (>99% yield, 0.5 mol% Pd), occurs under mild conditions (37 °C, aqueous media) and can be accelerated within biocompatible micelles at the cell membrane to yield products containing challenging biaryl bonds. This work highlights how native metabolic processes in anaerobic bacteria can be combined with green chemical technologies to produce highly efficient catalytic reactions for use in sustainable organic synthesis.

## Abstract 2

Nano-micelles meet biogenic Pd nanoparticles at the cell membrane promoting Suzuki Miyaura coupling reactions under mild conditions.

## Graphical abstract

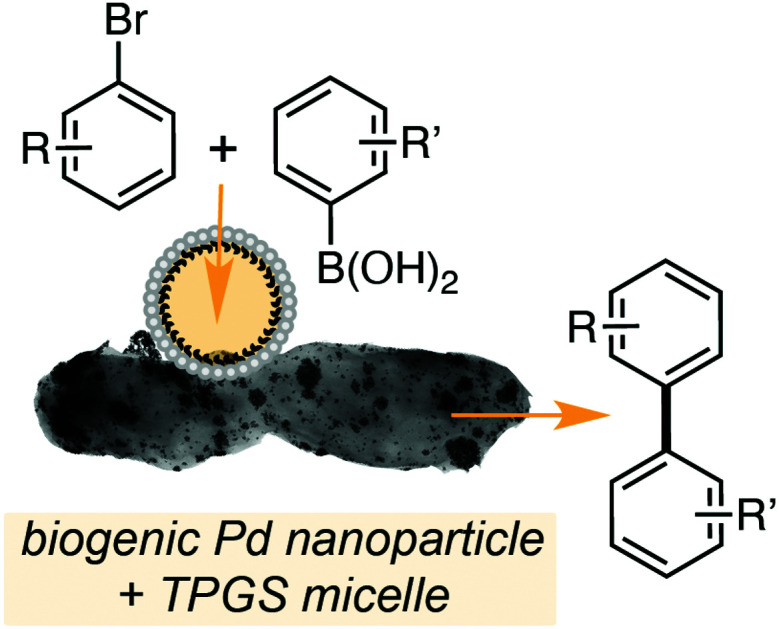

## Introduction

**Scheme 1:**
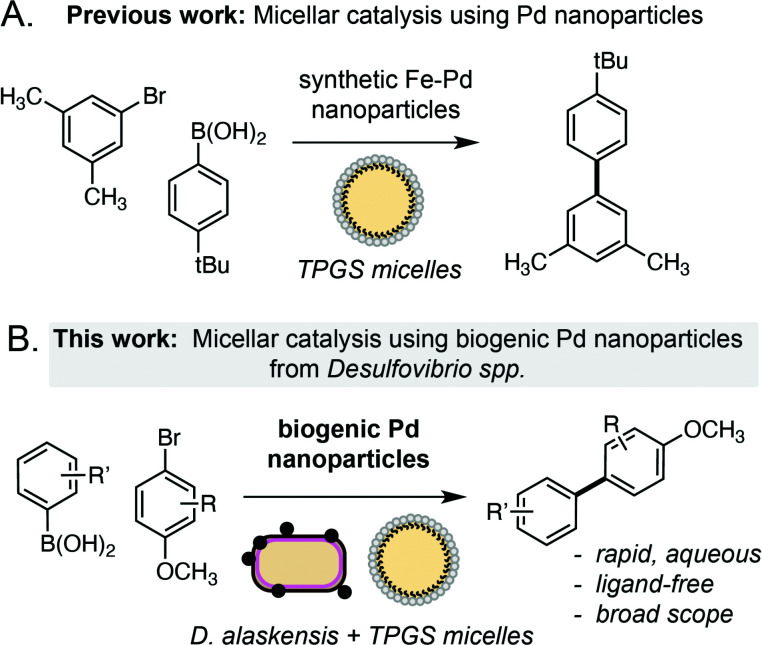
Approaches to the Suzuki Miyaura cross-coupling reactions in TPGS micelles using chemically synthesised or biogenic palladium nanoparticles.

Palladium is an important metal with unique chemical reactivity and a growing annual demand.^1^ However, the increasing scarcity and unsustainability of current recycling technologies (*e.g.* pyro/hydrometallurgy) has brought the remediation of Pd to the forefront of chemical research in recent years.^2^ Microorganisms offer an intriguing solution to this challenge, where native metal reduction pathways in bacteria can be used to convert soluble Pd waste into high-value metal nanoparticles.^3^ In particular, the sulfate-reducing bacterium *Desulfovibrio* spp. has emerged as an attractive choice owing to its remarkable tolerance to various metals^4^ and increasing genetic tractability.^5^ Reduction of soluble metals by *Desulfovibrio* spp. occurs in the periplasm and generates biogenic nanoparticles that are transported to the cell surface and tightly associated to the outer membrane.^6^ However, despite research into the mechanism^6*b*^ and scalability of this process for remediation purposes,^7^ the catalytic chemistry of metal nanoparticles generated by this microorganism is only now beginning to be explored. *Desulfovibrio* spp. has the unique capability to generate small nanoparticles in high yield (>95%) from a variety of feedstocks in the absence of external reductants. The biosynthesis of MNPs also offers distinct advantages over chemically synthesised equivalents from a product uniformity and sustainability viewpoint. Herein we report that biogenic Pd nanoparticles generated by *Desulfovibrio alaskensis* G20 are highly active catalysts for the ligand-free Suzuki Miyaura cross-coupling reaction under ambient conditions. The reaction has broad scope, can be dramatically accelerated within membrane associated TPGS micelles and outperforms other heterogeneous NP catalysts generated microbially or *via* chemical synthesis. Overall, this suggests a unique feature of the metal reduction pathway in *Desulfovibrio* spp. that is especially suited to the generation of highly active MNP catalysts for use in organic synthesis (Scheme 1).

## Results and discussion

Our studies began by investigating whether bacteriogenic Pd-NPs from *Desulfovibrio alaskensis* G20 (*Da*PdNPs) could catalyse C–C bond formation. We chose the Suzuki Miyaura reaction, inspired by previous studies by Deplanche *et al.*^8^ who demonstrated the use of *E. coli*-supported Pd-NPs to catalyse the cross-coupling of aryl halides and phenylboronic acid (62% conversion, EtOH_(aq)_, 80 °C). To this end, *Da*PdNPs were prepared from anaerobic cultures of *D. alaskensis* G20 grown in the presence of Na_2_PdCl_4_ (30 °C, 20 h) and isolated *via* centrifugation in 97% yield, as determined by ICP-OES. Analysis of the nanoparticles by X-ray diffraction (XRD) confirmed the presence of zero-valent Pd in a standard cubic arrangement (ESI, Fig. S2†). To test the reactivity of *Da*PdNPs we used the substrates 4-bromoanisole **1** and phenylboronic acid **2**. Reactions were carried out in deionised water at 37 °C for 20 h in the presence of ligands that are known to increase the reactivity of Pd in aqueous media. Encouragingly, 4-methoxybiphenyl **3** was detected in all reactions and also in the absence of any additional ligand (Table 1, entry 1). The ligand-free activity of biogenic MNPs at ambient temperature is rare and suggested to us that *Da*PdNPs possess unique catalytic properties. No product was observed using 4-chloroanisole and 4-iodoanisole showed no increase in reactivity when compared to **1**. Interestingly, the addition of triphenylphosphine decreased the cross coupling of **1** and **2** to 24%, whereas the bis-phenylphosphine ligands JohnPhos **4** and XPhos **6** typically used in Buchwald–Hartwig amination reactions increased the yield to 38% and 40%, respectively. The water-soluble ligands sSPhos **7** and AmPyol **8** decreased the yield 2–3-fold, despite being widely used to enhance the reactivity of Pd catalysed reactions in aqueous and/or biological conditions. However, the addition of tetramethylguanidine (TMG, **9**) increased the yield of **3** to 62%. As the cell membrane of *D. alaskensis* is negatively charged and tightly associates to Pd-NPs the co-localisation of cationic TMG to the cell surface under the reaction conditions could account for this observation. No further improvement in yield was observed using green tea polyphenols, despite the use of these plant-derived compounds being reported to enable ligand-free cross coupling reactions under aqueous conditions.^9^ Finally, we examined the mechanism of the reaction and compared the activity of biogenic nanoparticles to chemically synthesised equivalents. We confirmed the reaction is heterogeneous using a three-phase test by observing no product formation when using polymer-supported iodobenzoic acid (Table 1, entry 10), eliminating the possibility that catalysis occurs *via* the leaching of soluble Pd from *Da*PdNPs into solution. Interestingly, **3** was formed in <1% yield when using chemically synthesised heterogeneous Pd catalysts under the same reaction conditions. For example, Pd nanoparticles generated by chemical vapor deposition (cPdNP) and Pd on activated carbon (Pd/C) both afforded **3** in <1% yield, increasing to 5% for Pd/C in the presence of **9** (Table 1, entries 11–16). This intriguing result suggests a unique feature of nanoparticle biosynthesis in *D. alaskensis* that is especially suited to the generation of highly active heterogeneous Pd catalysts.

**Table 1:** Catalyst, ligand and additive screen for the DaPdNP catalysed Suzuki Miyaura reaction^a^

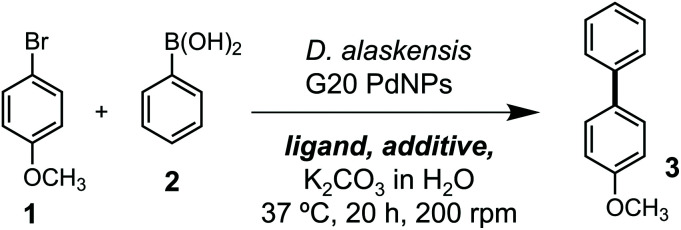				
Entry	Catalyst	Ligand	Additive	Yield (% ±SD)
1	*Da*PdNP	—	—	34
2	*Da*PdNP	PPh_3_	—	24
3	*Da*PdNP	JohnPhos	—	38
4	*Da*PdNP	SPhos	—	33
5	*Da*PdNP	XPhos	—	40
6	*Da*PdNP	sSPhos	—	6
7	*Da*PdNP	AmPyol	—	19
8	*Da*PdNP	TMG	—	62
9	*Da*PdNP	—	Green tea polyphenols	26
10^b^	*Da*PdNP	—	—	0
11	cPdNP	—	—	<1
12	cPdNP	XPhos	—	<1
13	cPdNP	TMG	—	<1
14	Pd/C	—	—	<1
15	Pd/C	XPhos	—	2
16	Pd/C	TMG	—	5
17	*Da*PdNP	—	TPGS-750-M^c^	75
18	*Da*PdNP	—	TPGS-1000^c^	>99

a Reactions were performed using **1** (25 mM), **2** (30 mM), K_2_CO_3_ (30 mM), Pd catalyst (0.25 mM) and ligand (2.5 mM) in sealed tubes under an atmosphere of air. Product concentrations were determined by ^1^H NMR spectroscopy relative to an internal standard of TMB. All data shown is an average of three experiments to one standard deviation.

b Polymer-supported iodobenzoic acid was used.

c 2% w/vol.

**Fig. 1:**
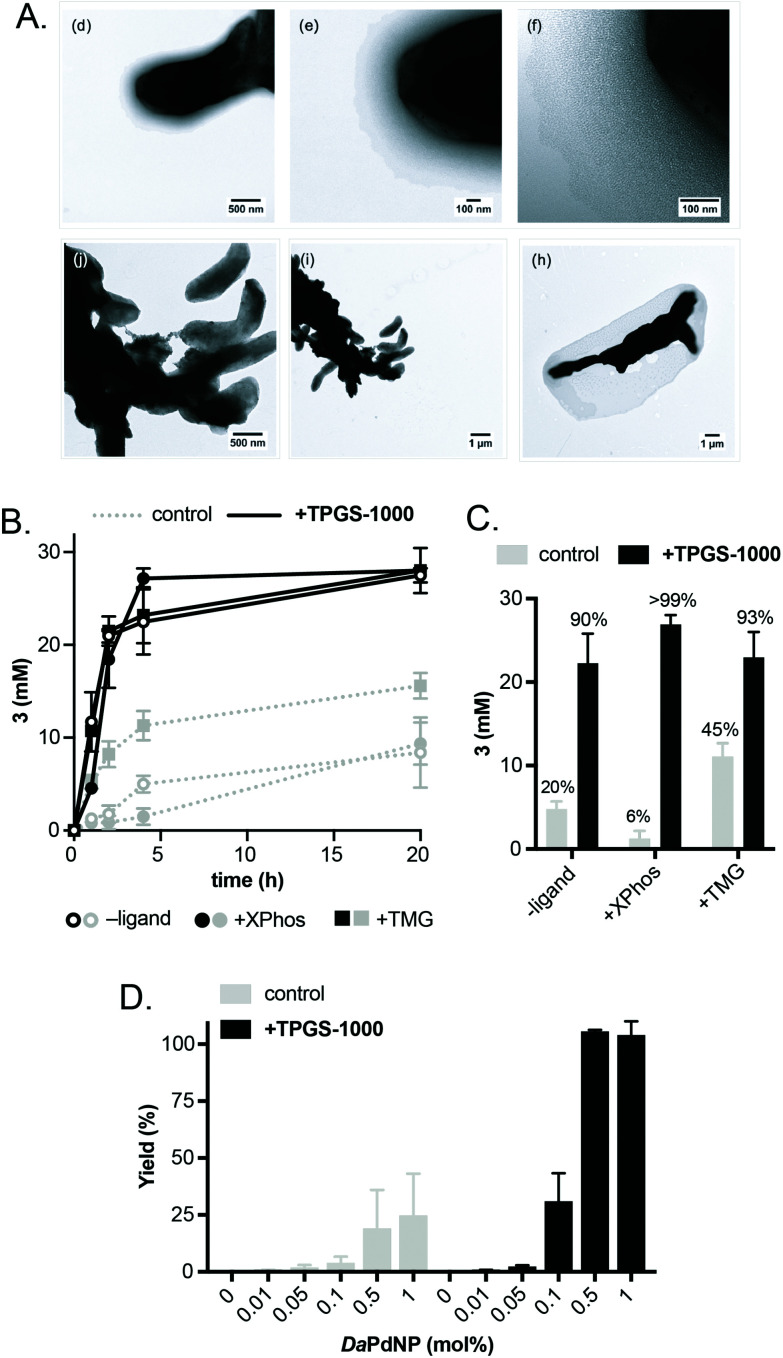
Examining the effect of TPGS micelles on the reaction. (A) TEM images of *Da*PdNPs in the presence and absence of TPGS-1000. (B) Time-course analysis showing the production of **3** and the effect of micelle addition. (C) Concentration of **3** in reactions after 4 h. The % conversion to **3** is shown above each dataset. (D) The effect of micelles on Pd activity at low catalyst loading. 2% w/v TPGS was added in all cases. Pd concentrations were determined by ICP-OES. Error bars represent the standard deviation of values from three independent experiments.

**Scheme 2:**
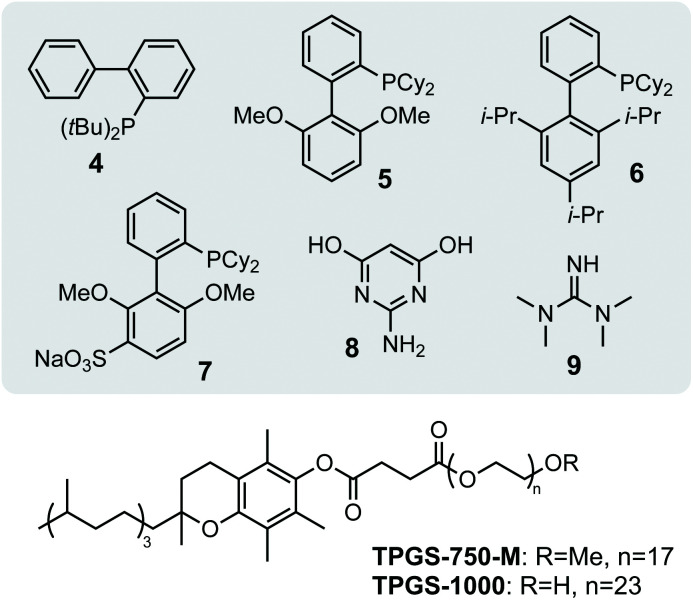
Structures of *P*-/*N*-ligands and surfactants used in screening studies. JohnPhos **4**, SPhos **5**, XPhos **6**, sSPhos **7**, AmPyol **8**, TMG **9**.

Having confirmed the activity of *Da*PdNPs under aqueous conditions we moved on to examine methods to increase their activity *in vivo*. To begin, we examined the effect of co-localising the reaction within TPGS micelles. These vitamin E-derived surfactants are known to self-assemble in aqueous solution and promote organic reactions by co-localising reactants in the hydrophobic micelle interior.^10^ They also associate with cell membranes and accelerate flux through engineered metabolic pathways by sequestering hydrophobic metabolites.^11^ Therefore, as Pd nanoparticles associate to the outer membrane of *D. alaskensis*, we hypothesised that co-localisation of **1** and **2** to this region would also accelerate product formation. To our delight, the addition of 2% w/v TPGS-750-M and TPGS-1000 significantly increased the reactivity of *Da*PdNPs in the absence of ligand, affording **3** in 75% and >99% yield, respectively. Increased product formation was observed for all combinations of surfactant and ligand, except for the use of **4** and TPGS-750-M (ESI, Table S3,† entry 3). In all cases, the hydroxylated surfactant TPGS-1000 outperformed the *O*-methylated congener TPGS-750-M. To explore the reasons for this, we examined cells by transmission electron microscopy (TEM). Interestingly, the addition of TPGS-1000 produced highly-ordered micelles at the cell surface (Fig. 1A and ESI, Fig. S7†), whereas TPGS-750-M produced disordered agglomerates that appeared to disrupt cell morphology (ESI, Fig. S8†). Although the increased membrane penetration of TPGS-750-M has been observed in *E. coli* and attributed to the increased hydrophobicity of the micelle surface (Scheme 2, R = CH_3_, *n* = 17), this was shown to have no effect on the rate of reactions in the micelle interior.^11^ For *D. alaskensis*, however, TPGS-750-M micelles embedded in the cell membrane are distanced from MNPs bound at the cell surface and could therefore explain the reduced yield of **3**. Extracellular presentation of *Da*PdNPs could also combine with favourable hydrogen bonding interactions between TPGS-1000 (Scheme 2, R = H, *n* = 23) and outer-cell matrix polysaccharides surrounding the Pd nanoparticles to co-localise the substrates and catalyst. Altogether, this provides evidence to support the hypothesis that TPGS-1000 and TPGS-750-M enhance the reactivity of biogenic Pd nanoparticles from *D. alaskensis* by localising the reaction components at the outer cell membrane. The precise nature of this interaction and the generality of this effect across various microorganisms is underexplored and currently under investigation by our laboratories. To further assess the extent to which TPGS micelles accelerate the reaction we moved on to measure product formation over time (Fig. 1B). In the absence of TPGS-750-M or TPGS-1000, the reaction reached 40 and 60% conversion after 20 h in the presence of ligand **6** or **9**, respectively. In contrast, TPGS-1000 accelerated the formation of **3** to 90% after 4 h in the absence of ligand and to >99% and 93% yield using **6** and **9**, respectively (Fig. 1C). High reactivity was also maintained at low catalyst loading. For example, reducing *Da*PdNPs to 1 mol% reduced product conversion to 25% in the absence of micelles, whereas quantitative conversion was observed in the presence of TPGS-1000 at catalyst loadings of ≥0.5 mol% (Fig. 1D). This reduced to 31% and 4% yield at 0.1 mol% Pd in the presence and absence of TPGS-1000, respectively. Overall, this dramatic effect (>5-fold) highlights the unique benefits of micellar catalysis in *D. alaskensis* cells and how this can be combined with organic ligands designed for use in synthetic chemistry to enhance the reactivity of biological metals *in vivo*.

**Fig. 2:**
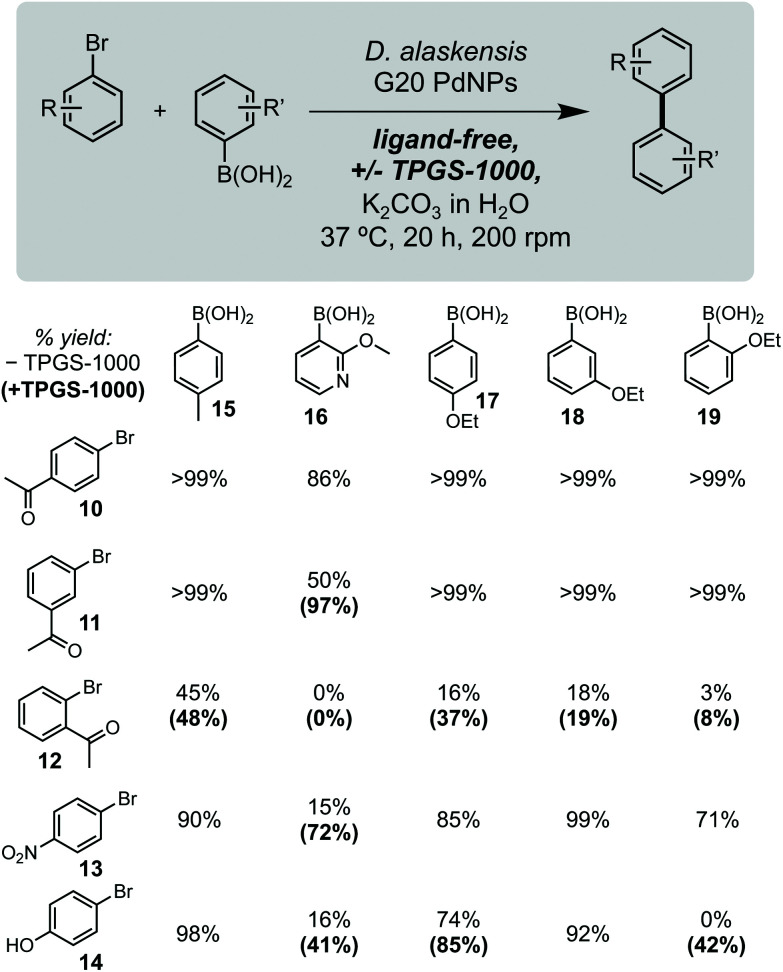
Substrate scope. Yields in parenthesis are from reactions containing 2% w/v TPGS-1000. Product concentrations determined by ^1^H NMR spectroscopy relative to an internal standard of TMB (10 mM).

Using these optimised conditions, we next investigated the reaction scope using a range of aryl bromide and aryl boronic acid substrates (Fig. 2). This included arenes containing a range of electron withdrawing and donating function groups at *ortho*-, *meta*- and *para*-positions and included the heterocyclic (methoxy)pyridyl boronic acid (MeOPyB, **16**). To our delight, *Da*PdNPs were effective catalysts for most substrates tested and when combined with TPGS-1000 enabled the formation of challenging sp^2^ C–C bonds. Electron withdrawing acyl groups were tolerated at all positions in the aryl bromide substrate. Reactivity was reduced for *ortho*-acyl and *ortho*-ethoxy substrates **12** and **19** due to steric hindrance, but could be increased through the addition of TPGS-1000. For the cross coupling of **11** and **16**, **12** and **17**, **12** and **19**, **13** and **16** and **14** and **16**, the use of TPGS-1000 increased product yield >2-fold. This is particularly impressive for heteroaromatic substrates and the cross-coupling of two *ortho*-substituted aromatics. Notably, **14** and **19** were unreactive in the presence of *Da*PdNPs but product formation was increased to 42% in the presence of TPGS-1000 micelles. Overall, this shows that TPGS micelles not only accelerate product formation in the presence of cells but also increase the overall reaction scope of biogenic PdNPs.

## Conclusions

In summary, we have demonstrated that Pd^0^ nanoparticles synthesised by *Desulfovibrio alaskensis* are highly active heterogeneous catalysts for the Suzuki Miyaura reaction of aryl bromides and phenylboronic acids. These biological catalysts can be readily prepared from bacterial cell culture using common Pd salts in high yield (>97%) and outperform other available heterogenous Pd catalysts generated *via* chemical or biological methods. We show that reactions catalysed by these nanoparticles can be enhanced using organic ligands and/or designer micelles to co-localise substrates at the cell membrane. The combination of organic chemistry and microbiology tools results in an overall reaction that is highly efficient (>99% yield, 0.5 mol% Pd), occurs under ambient conditions (aqueous media, 37 °C) and can be applied to a wide range of substrates to generate products containing challenging sp^2^ C–C bonds. To the best of our knowledge, this is the first report of a ligand-free Suzuki Miyaura reaction catalysed by a biological Pd catalyst in micellar nanoreactors. Future studies will focus on genetically engineering *D. alaskensis* to produce “designer NPs” with increased reactivity and extending this process to encompass the use of industrial waste streams as a metal resource.

## Author contributions

L. E. H. and S. W. conceived and directed the project. Y. E. and J. A. D. planned and conducted experimental work. All authors contributed to discussions and manuscript writing.

## Conflicts of interest

There are no conflicts to declare.

## Supplementary Material

GC-023-D1GC02392F-s001
