# Supplementary material for: Micellar catalysis of the Suzuki Miyaura reaction using biogenic Pd nanoparticles from Desulfovibrio alaskensis
Source: Green Chem. 2021 Oct 11;23(22):8886–90. doi: 10.1039/d1gc02392f (PMC8593813; doi:10.1039/d1gc02392f)
Supplement: GC-023-D1GC02392F-s001 [file GC-023-D1GC02392F-s001.pdf]

Electronic Supplementary Material (ESI) for Green Chemistry.
This journal is © The Royal Society of Chemistry 2021

SUPPLEMENTARY INFORMATION

Micellar catalysis of the Suzuki Miyaura reaction using biogenic Pd nanoparticles from *Desulfovibrio alaskensis*

Yuta Era, Jonathan A. Dennis, Stephen Wallace* and Louise E. Horsfall*

Institute of Quantitative Biology, Biochemistry and Biotechnology, School of Biological Sciences, University of Edinburgh, Roger Land Building, Alexander Crum Brown Road, King's Buildings, Edinburgh, EH9 3FF

*Correspondence to: louise.horsfall@ed.ac.uk; stephen.wallace@ed.ac.uk

Supplementary Materials

S1 General materials and methods S2

S2 Strain, media and culture condition S3

S3 *Da*PdNPs production by *Desulfovibrio alaskensis* S3

Fig. S1 *Representative Electron micrographs of DaPdNPs*

Fig. S2 *XRD pattern of the DaPdNPs synthesised by D. alaskensis*

Fig. S3 *ICP standard curve*

S4 General Suzuki coupling reaction S5

Fig. S4 *Representative ^1H NMR spectra*

Fig. S5 *Result of control experiments*

S5 Coupling reaction optimisation in aqueous solution S7

Table S1 *Ligand/additive and halides screen*

S6 Coupling reaction with chemically produced Pd catalysts S8

Table S2 *Coupling reaction promoted by chemically made Pd catalysts*

S7 Micellar coupling reaction in the presence of various ligands S8

Table S3 *Micellar coupling reaction in the presence of various ligands*

S8 Effect of TPGS-1000 on the reaction speed and the catalyst loading minimisation S9

Table S4 *Time-course experiments and catalyst loading optimisation without TPGS-1000*

S9 Investigation of the reaction scope S10

Table S5 *Reaction scope*

S10 Three-phase test S13

Fig. S6 *Three-phase test*

Table S6 *Three-phase test*

S11 TEM analysis of *Da*PdNPs and TPGS micelles S14

Fig. S7 *Negative stained DaPdNPs in the presence of 0.4% w/v TPGS-1000*

Fig. S8 *Negative stained DaPdNPs in the presence of 0.4% w/v TPGS-750-M*

S12 Product and Pd separation S17

Fig. S9 *Pelleted DaPdNPs during liquid-liquid extraction*

S13 Product characterisation S18

S14 References S25

S1 General materials and methods

Unless otherwise noted, starting materials and reagents were obtained from commercial suppliers and were used without further purification. All water used experimentally was purified with a Suez Select purification system (18 mΩ/cm, 0.2 μM filter).

NMR and IR: Proton nuclear magnetic resonance spectra (^{1}H NMR) were recorded using an AVA 500 NMR spectrometer (Bruker) at the specified frequency at 298K. Proton chemical shifts are expressed in parts per million (ppm, δ scale) and are referenced to residual protium in the NMR solvent ($CDCl_3$, ∂ 7.26 ppm). Coupling constants, *J*, are measured to the nearest 0.1 Hz and are presented as observed. Data is represented as: chemical shift, integration, multiplicity (s = singlet, d = doublet, t = triplet, q = quartet, dd = doublet of doublet, m = multiplet and/or multiple resonances), coupling constant (J) in Hertz. NMR solvents were used as purchased from commercial suppliers. For all quantitative NMR measurements, 1,3,5-trimethoxybenzene (TMB) was used as an internal standard. Infrared (IR) spectroscopy was performed using a Spectrum Two IR spectrometer (Perkin Elmer).

XRD: X-ray diffraction experiments were recorded on a MiniFlex 600 benchtop X-ray diffractometer (Rigaku) with D/teX Ultra 1D silicon strip detector (Rigaku) using Cu$K\alpha$ radiation. Scanning was run at a 2θ angle from 20 to 100° with a 2° step size (40 kV, 15 mA).

ICP-OES: Inductively Coupled Plasma Optical Emission Spectrometry (ICP-OES) was performed using an Optima 8300 instrument (Perkin Elmer). Experiments were performed using the following conditions: samples were sonicated for 30 min at 21 ˚C in a water bath and then centrifuged (20,000 × g, 2 h). The supernatant (the ionic fraction) was added to a solution of aqua regia ($HCl:HNO_3$ = 14:10%) and heated to 80°C for 8 h. The resulting sample was diluted in 2% nitric acid to a final volume of 3 mL and used immediately. Unspun samples (whole fraction) were prepared using an analogous method. When quantifying by ICP a standard curve was constructed over a range of analyte concentrations (0.04–10 ppm) providing linear relationships of Intensity = 41356c_{Pd} + 1573.4.

TEM: Transmission electron microscopy (TEM) was performed using a JEM-1400 Plus (JEOL) with an accelerating voltage of 80 kV. TEM images were captured using a GATAN OneView camera. Image processing was carried out using ImageJ software. Experiments were performed using the following conditions: suspensions of *Da*PdNPs were drop cast on to a 200 mesh carbon-coated copper grid and dried for 5 min under air. The excess liquid was then removed and the samples visualised immediately. using a TEM (JEM-1400 Plus, JEOL) with an accelerating voltage of 80 kV.

S2 Strain, media, buffer and culture condition

Desulfovibrio alaskensis G20 (DSM 17464) was obtained from DSMZ. *Desulfovibrio alaskensis* G20 (DSM 17464) was grown statically on de-gassed Postgate Medium C at 30 °C in an anaerobic chamber fed with 10% H_2 and 10% CO_2 in nitrogen. Optical densities of *D. alaskensis* cultures were determined using a WPA CO8000 spectrophotometer (Biochorm) by measuring absorbance at 600 nm.

Postgate Medium C was prepared according to the following procedure: KH_2PO_4 (0.5 g), NH_4Cl (1.0 g), Na_2SO_4 (4.5 g), $CaCl_2 \cdot 6H_2O$ (0.06 g), $MgSO_4 \cdot 7H_2O$ (0.06 g), 60% sodium lactate (10 mL) yeast extract (1.0 g), $FeSO_4 \cdot 7H_2O$ (0.004 g) and sodium citrate$\cdot 2H_2O$ (0.3 g) were dissolved in 990 mL ultrapure water and its pH was adjusted to 7.5 using 2 M NaOH. The medium was autoclaved at 121 °C for 20 min and cooled to room temperature. Autoclaved Postgate Medium C was stored at room temperature and degassed in the anaerobic chamber overnight before the use.

MOPs buffer was prepared according to the following procedure: MOPs free acid (41.86 g) was dissolved in 1 L ultrapure water and its pH was adjusted to 7.0 using 1 M H_2SO_4. The buffer was autoclaved at 121 °C for 20 min and cooled to room temperature. Autoclaved MOPs buffer was stored at room temperature and degassed in the anaerobic chamber overnight before the use.

S3 *Da*PdNPs production by *Desulfovibrio alaskensis*

D. alaskensis cells were pre-grown following previously reported methods[1], recovered and washed with MOPs buffer three times prior to inoculation (to a final OD_{600} of 1.0) in 50 mL centrifuge tubes containing 40 mL MOPs buffer. Freshly made Na_2PdCl_4 stock solution (40 mM in H_2O) was added to the cell suspension to a final concentration of 2 mM. The centrifuge tubes were incubated at 30 ˚C for 20 h in an anaerobic chamber. The biogenic nanoparticles (*Da*PdNPs) were harvested by centrifugation at 15 min, 4,500 × g) and washed with 50% acetone in H_2O (1 x vol equiv.). Subsequently, the *Da*PdNPs were freeze-dried overnight, resuspended in ultrapure water and sonicated in a water bath for 30 min. The resulting nanoparticles were analysed by XRD and TEM. The recovery of Pd as *Da*PdNPs was determined by ICP-OES.

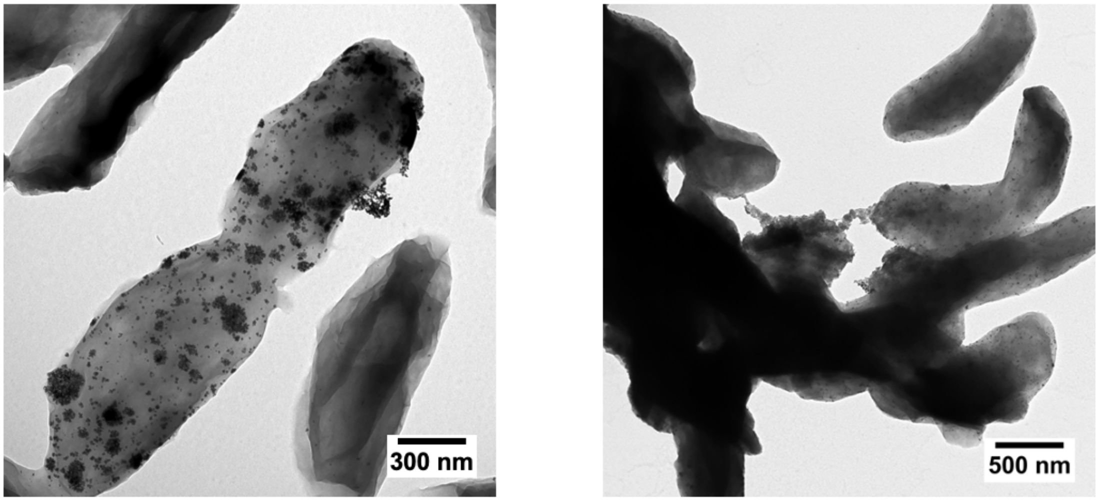

Fig. S1 *Representative Electron micrographs of DaPdNPs.*

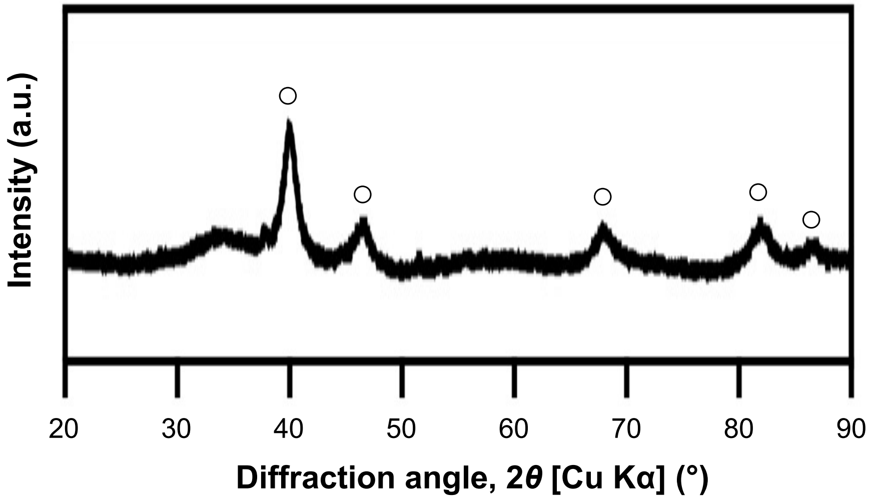

Fig. S2 *XRD pattern of the DaPdNPs synthesised by D. alaskensis. The symbol ○ is assigned to palladium (JCPDS 87-0641).*

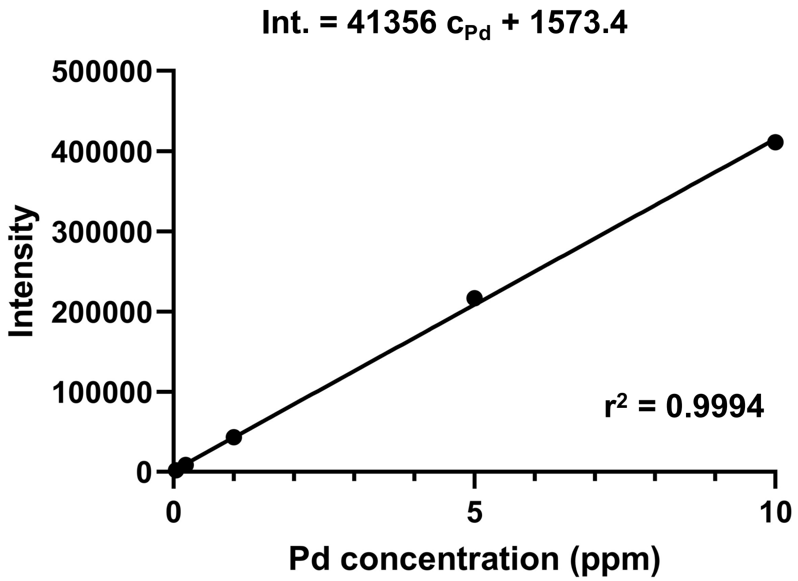

Fig. S3 *ICP standard curve used for the quantification of palladium recovered as DaPdNP. DaPdNP was ionised with aqua regia prior to the analysis.*

S4 General Suzuki coupling reaction

Cross coupling reactions were carried out using the following procedure: *Da*PdNPs were added to a 15 mL Hungate tube containing aryl halide (25 mM), phenyl boronic acid (30 mM), K_2CO_3 (30 mM) and TPGS-1000/TPGS-750-M (2% w/v) in H_2O (5 mL). The tubes were sealed with butyl rubber septa and screw-caps and incubated at 37 ˚C (200 rpm) for 20 h in a New Brunswick Innova 44 incubator shaker (Eppendorf). After this time, the reactions were cooled to room temperature, extracted with methyl *tert*-butyl ether (MTBE, 3 x 1.7 mL) and concentrated under reduced pressure. The crude residue was dissolved in 1 mL $CDCl_3$ containing 10 mM TMB and analysed by ^1H NMR spectroscopy.

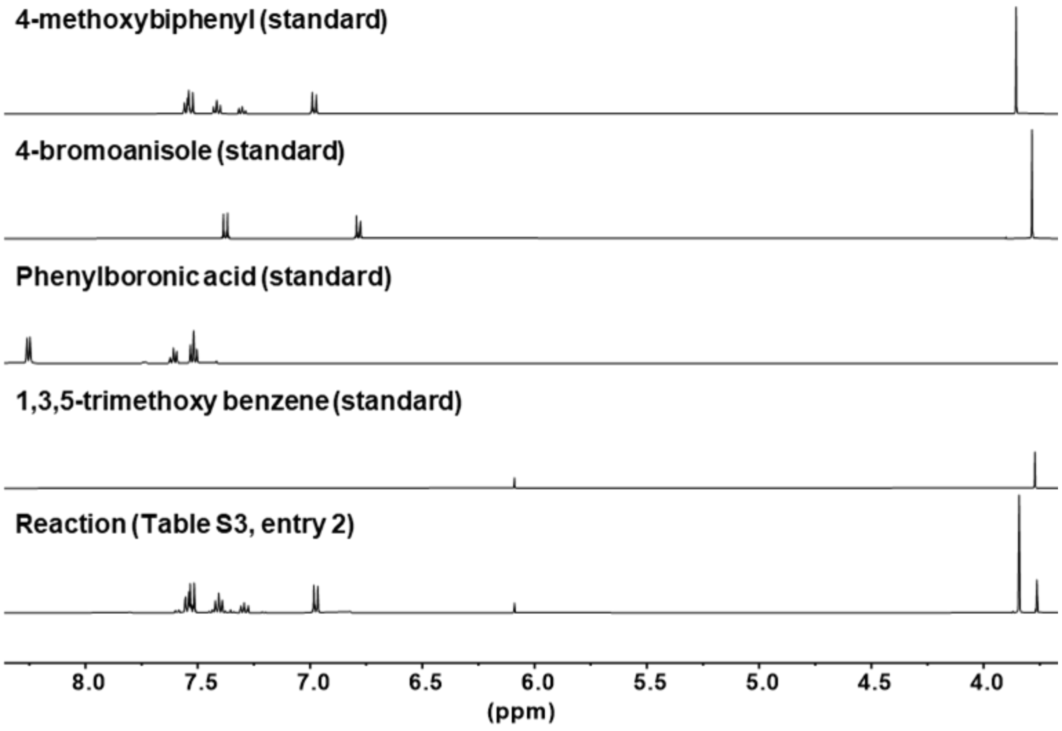

Fig. S4 *Representative ^{1}H NMR spectra.*

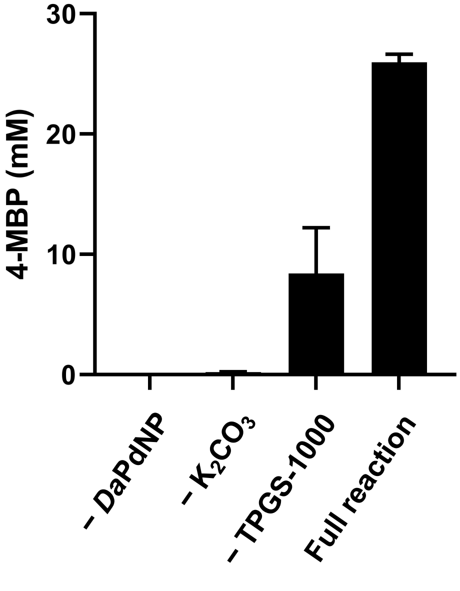

Fig. S5 *Result of control experiments. Full reaction was conducted using DaPdNPs (0.25 mM), 4-bromoanisole (25 mM), phenylboronic acid (30 mM), K_2CO_3 (30 mM) and TPGS-1000 (2% w/v) in H_2O (5 mL).*

S5 Coupling reaction optimisation in aqueous solution

Unless otherwise noted, model reaction (*Da*PdNPs (0.25 mM), 4-bromanisole (25 mM), phenylboronic acid (30 mM), K_2CO_3 (30 mM) in H_2O (5 mL)) was used by following the above protocol for reaction optimisation and screening experiments. For ligand/additive screening, triphenylphosphine (PPh_3), JohnPhos, SPhos, XPhos, sSPhos, 4-aminopyridine-2,6-diol (AmPyol), 1,1,3,3-tetramethylguanidine (TMG) or green tea polyphenols was added to the model reaction (2.5 mM for ligands and 0.1/ or 0.01 % w/v for green tea polyphenols). For halide screening, 4-chloroanisole/4-iodoanisole (25 mM) was used as the coupling partner instead of 4-bromoanisole.

Table S1 *Ligand/additive and halides screen*

#	Ar-X	Ar-B(OH)2	Ligand	*Da*PdNPs loading (mol%)	Additive	Yield	
						mM	±SD
1			–			8.4	3.8
2			PPh_3			6.0	0.3
3			JohnPhos			9.0	1.1
4			SPhos			8.1	1.9
5			XPhos		–	14.0	1.8
6	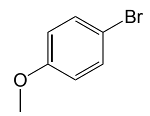		TMG			15.6	1.4
7			AmPyol	1.0		5.0	0.5
8			sSPhos			1.6	0.2
9		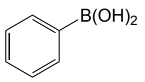	–		Green tea polyphenols (0.1% w/v)	0.4	0.1
10			–		Green tea polyphenols (0.01% w/v)	6.4	0.5
11	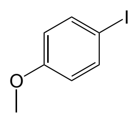		–	1.0	–	9.0	0.5
12	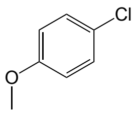		–	1.0		0.2	0.0

The yield shown above is the average of triplicate experiments.

S6 Coupling reaction with chemically produced Pd catalysts

Chemically produced Pd nanoparticle (cPdNP, 0.25 mM) or Pd on activated carbon (Pd/C, 0.25 mM) was added to the model reaction with/without ligands (XPhos or TMG, 2.5 mM) in place of *Da*PdNPs. The reactions were conducted and analysed by following the protocol in Section S4.

Table S2 *Coupling reaction promoted by chemically made Pd catalysts*

#	Ar-X	Ar-B(OH)$_2$	Ligand	Pd catalyst	Additive	Yield mM	Yield ±SD
1	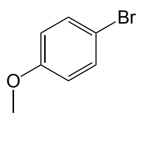	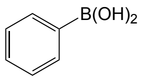	–	cPdNPs	–	0.2	0.2
2			XPhos			0.2	0.0
3			TMG			0.3	0.2
4			–	Pd/C		0.1	0.0
5			XPhos			1.3	0.5
6			TMG			0.4	0.7

The yield shown above is the average of triplicate experiments.

S7 Micellar coupling reaction in the presence of various ligands

The model reaction containing no ligand, JohnPhos, SPhos, XPhos or TMG (2.5 mM) was conducted in the presence of TPGS-750-M or TPGS-1000 (2% w/v). The reactions were conducted and analysed by following the protocol in Section S4.

Table S3 *Micellar coupling reaction in the presence of various ligands*

#	Ar-X	Ar-B(OH)$_2$	Ligand	*Da*PdNPs loading (mol%)	Additive	Yield mM	Yield ±SD
1	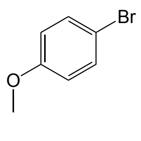	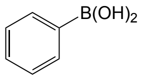	-	1.0	TPGS-750-M	20.8	1.4
2					TPGS-1000	>25	0.7
3			JohnPhos		TPGS-750-M	11.1	1.0
4					TPGS-1000	22.2	1.0
5			SPhos		TPGS-750-M	12.0	0.9
6					TPGS-1000	18.3	0.7
7[a]			XPhos		TPGS-750-M	21.5	2.1
8					TPGS-1000	>25	2.2
9			TMG		TPGS-750-M	19.3	0.9
10					TPGS-1000	24.9	1.0

The yield shown above is the average of triplicate experiments ([a]: duplicate).

S8 Effect of TPGS-1000 on the reaction speed and the catalyst loading minimisation

Reactions containing TPGS-1000 (2% w/v) or no surfactant and either no ligand, XPhos or TMG (2.5 mM) was sealed and incubated at 37 ˚C (200 rpm) in a New Brunswick Innova 44 incubator shaker (Eppendorf). Reactions were collected at 1, 2, 4, 20 and 40 h, and extracted with extracted with MTBE (3 x 1.7 mL) immediately. For the catalyst loading minimisation experiment, the concentration of *Da*PdNPs was adjusted to 0.01, 0.05, 0.1, 0.5 or 1.0 in the ligand-free model reaction with/without TPGS-1000 (2% w/v). The reactions and catalyst-free control reaction were sealed and incubated at 37 ˚C (200 rpm) for 20 h. The following analysis was carried out as outlined in Section S4.

Table S4 *Time-course experiments and catalyst loading optimisation without TPGS-1000*

#	Ar-X	Ar-B(OH)$_2$	Ligand	Reaction time (h)	*Da*PdNPs loading (mol%)	Additive	Yield	
							mM	±SD
1	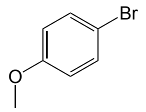	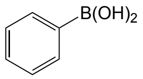	–	1	1.0	–	1.3	0.4
2				2			1.8	0.9
3				4			5.0	0.9
4				20			8.4	3.8
5				40			7.0	4.8
6				1		TPGS-1000	11.7	3.2
7				2			21.0	0.7
8				4			22.5	3.5
9				20			>25	0.8
10				40			>25	1.0
11	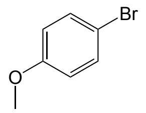	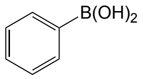	XPhos	1	1.0	–	0.8	0.2
12				2			0.9	0.7
13				4			1.5	0.9
14				20			9.4	2.3
15				40			6.7	1.4
16				1		TPGS-1000	4.6	0.4
17				2			18.4	3.1
18				4			>25	1.1
19				20			>25	0.5
20				40			>25	2.0
21	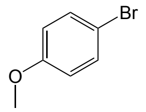	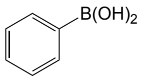	TMG	1	1.0	–	5.2	0.7
22				2			8.2	1.4
23				4			11.3	1.6
24				20			15.6	1.4
25				40			14.8	2.6

26				1		TPGS-1000	10.7	0.6
27				2			21.5	1.6
28				4			23.2	3.0
29				20			>25	2.4
30				40			>25	1.2
31	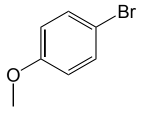	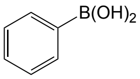	–	20	0	–	N.D.	N.A.
32					0.01		0.2	0.2
33					0.05		0.5	0.3
34					0.1		1.0	0.7
35					0.5		4.7	4.3
36					1.0		6.2	4.5
37				20	0	TPGS-1000	N.D.	N.A.
38					0.01		0.1	0.1
39					0.05		0.6	0.2
40					0.1		7.7	3.2
41					0.5		>25	0.2
42					1.0		>25	1.5

The yield shown above is the average of triplicate experiments (N.D.: Not detected, N.A.: Not applicable).

S9 Investigation of the reaction scope

Aryl bromide (4-bromoacetophenone, 3-bromoacetophenone, 2-bromoacetophenone, 1-bromo-4-nitrobenzene or 4-bromophenol: 25 mM) and boronic acid (*p*-tolylboronic acid, 2-methoxy-3-pyridinylboronic acid, 4-ethoxyphenylboronic acid, 3-ethoxyphenylboronic acid, 3-ethoxyphenylboronic acid :30 mM) were added to Hungate tube containing *Da*PdNPs (0.25 mM) and K_2CO_3 (30 mM) in H_2O (5 mL). Reactions of 25 different cross-coupling combination were carried out and analysed as outlined in Section S4. Subsequently, for the reactions resulting in less than 80% yield, the identical coupling reactions were conducted in the presence of TPGS-1000 (2% w/v).

Table S5 *Reaction scope*

#	Ar-X	Ar-B(OH)₂	Ligand	*Da*PdNPs loading (mol%)	Additive	Yield	
						mM	±SD
1		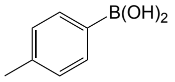			–	>25	1.5
2		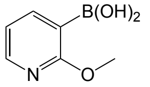			–	21.6	1.7
3	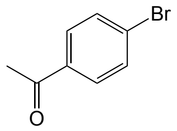	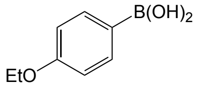	–	1.0	–	>25	1.5
4		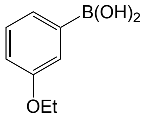			–	>25	0.2
5		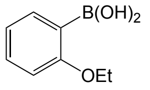			–	>25	1.1
6		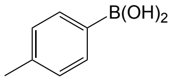			–	>25	0.6
7		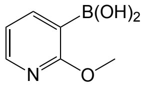			–	12.5	5.3
8[b]					TPGS-1000	24.3	N.A.
9	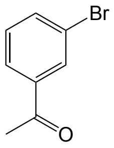	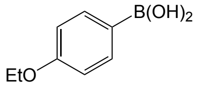	–	1.0	–	>25	1.5
10		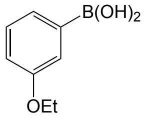			–	>25	1.3
11		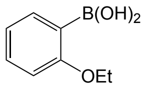			–	>25	1.5

12[a]	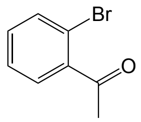	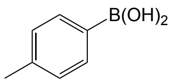	–	1.0	–	11.3	0.5
13[a]					TPGS-1000	12.1	3.9
14		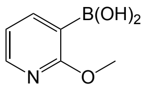			–	N.D.	N.A.
15					TPGS-1000	N.D.	N.A.
16[a]		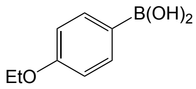			–	4.1	0.3
17[a]					TPGS-1000	9.4	0.4
18		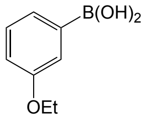			–	4.5	0.1
19					TPGS-1000	4.8	0.0
20		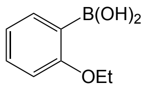			–	0.9	0.2
21					TPGS-1000	2.0	0.2
22	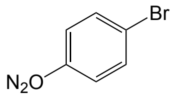	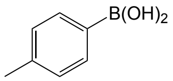	–	1.0	–	22.6	6.1
23[a]		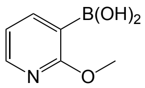			–	3.7	0.0
24[a]					TPGS-1000	18.1	0.2
25		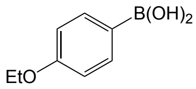			–	21.2	1.7
26		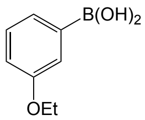			–	24.6	2.0
27[a]		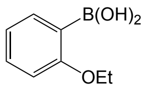			–	17.7	0.6
28[a]					TPGS-1000	18.8	0.7
29		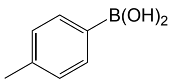	–	1.0	–	24.6	1.7

30	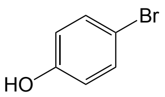	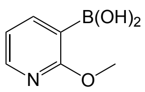	–	1.0	–	4.0	0.4
31[a]					TPGS-1000	10.2	0.6
32		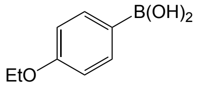			–	18.6	1.7
33					TPGS-1000	21.3	0.6
34		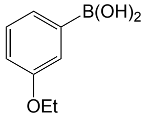			–	23.0	0.5
35[a]		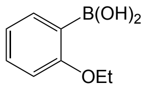			–	N.D.	N.A.
36[a]					TPGS-1000	10.6	0.7

The yield shown above is the average of triplicate experiments ([a]: duplicate, [b]: no replicate, N.D.: Not detected, N.A.: Not applicable).

S10 Three-phase test

For the solid support, the Wang resin, a polystyrene based resin with a p-benzyloxybenzyl alcohol linker often used in solid phase peptide synthesis was chosen[2]. Iodobenzoic acid bound to a Wang resin was synthesised according to the following procedure: Wang resin (2.0 g, 1.0 mmol/g loading) was added to a solution of dichloromethane:*N*,*N*-dimethylformamide (9:1, 20 mL) and stirred for 15 min. *N*, *N*'-diisopropylcarbodiimide (930 µL, 300 mM), 4-iodobenzoic acid (2.5 g, 500 mM) and 4-(dimethylamino)pyridine (1.0 mg, 0.4 mM) were added to the mixture and the reaction was stirred for 3 days at room temperature (21 °C). The resin was recovered by filtration, washed with dichloromethane (3 x 20 mL) and dried under vacuum. Formation of the target compound was confirmed by the appearance of a characteristic ester stretch in the IR spectrum of the product at 1615 cm^{-1}. For the three-phase test, polymer-supported iodobenzoic acid was added in place of haloaryls following standard cross-coupling reaction conditions. After incubation, the solid was collected by vacuum filtration, washed with dichloromethane (4 x 5.0 mL), dried under vacuum, dissolved in TFA:dichloromethane (9:1, 2.0 mL) and stirred at room temperature for 2 h. The resin was collected by vacuum filtration and washed with dichloromethane (4 x 5.0 mL). The combined organic layers were concentrated under reduced pressure. Control reactions were conducted using methyl 4-iodobenzoate (33 mg, 25 mM, 1.0 equiv) instead of the polymer-supported iodobenzoic acid and analysed according to the general procedure outlined in Section S4.

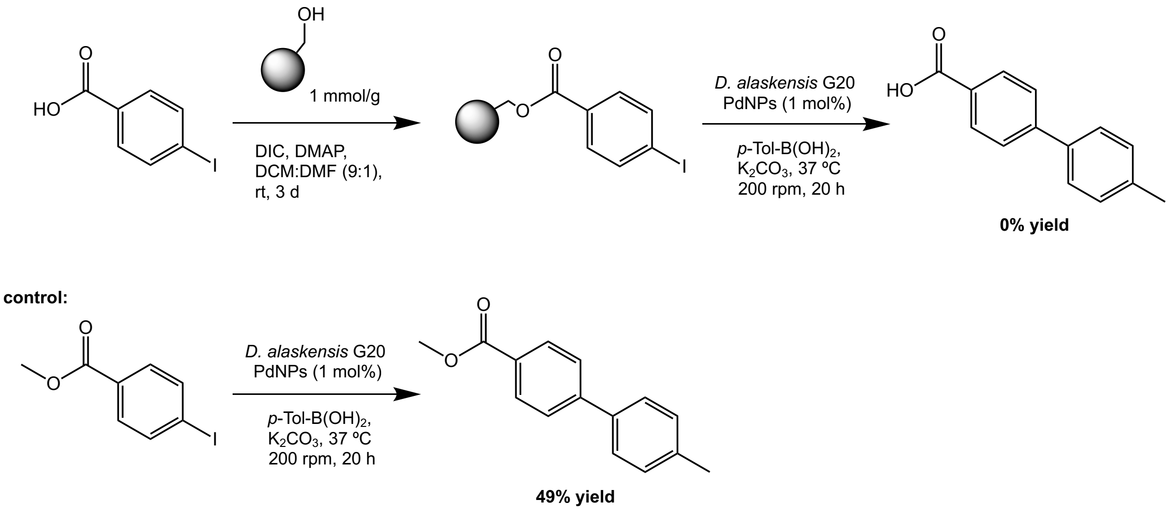

Fig. S6 *Three-phase test*

Table S6 *Three-phase test*

#	Ar-X	Ar-B(OH)$_2$	Ligand	*Da*PdNPs loading (mol%)	Additive	Yield	
						mM	±SD
1	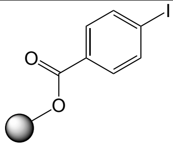	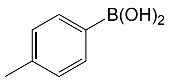	–	1.0	–	N.D.	N.A.
2	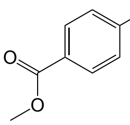					12.2	3.5

The yield shown above is the average of duplicate experiments (N.D.: Not detected, N.A.: Not applicable).

S11 TEM analysis of *Da*PdNPs and TPGS micelles

Samples were prepared for TEM analysis by incubating *Da*PdNPs (0.25 mM) and TPGS-1000 or TPGS-750-M (0.4% w/v) in H_2O at 37 ˚C (220 rpm) for 12 h. One drop was then transferred on to a Formvar/Carbon 200 mesh Copper grid and left to dry in air for 10 min. Excess solution was removed by touching the grid edge with filter paper. A drop of 1% aqueous uranyl acetate was applied for 1 minute and then removed by touching the grid edge with filter paper. TEM observation was carried out as described in Section S1.

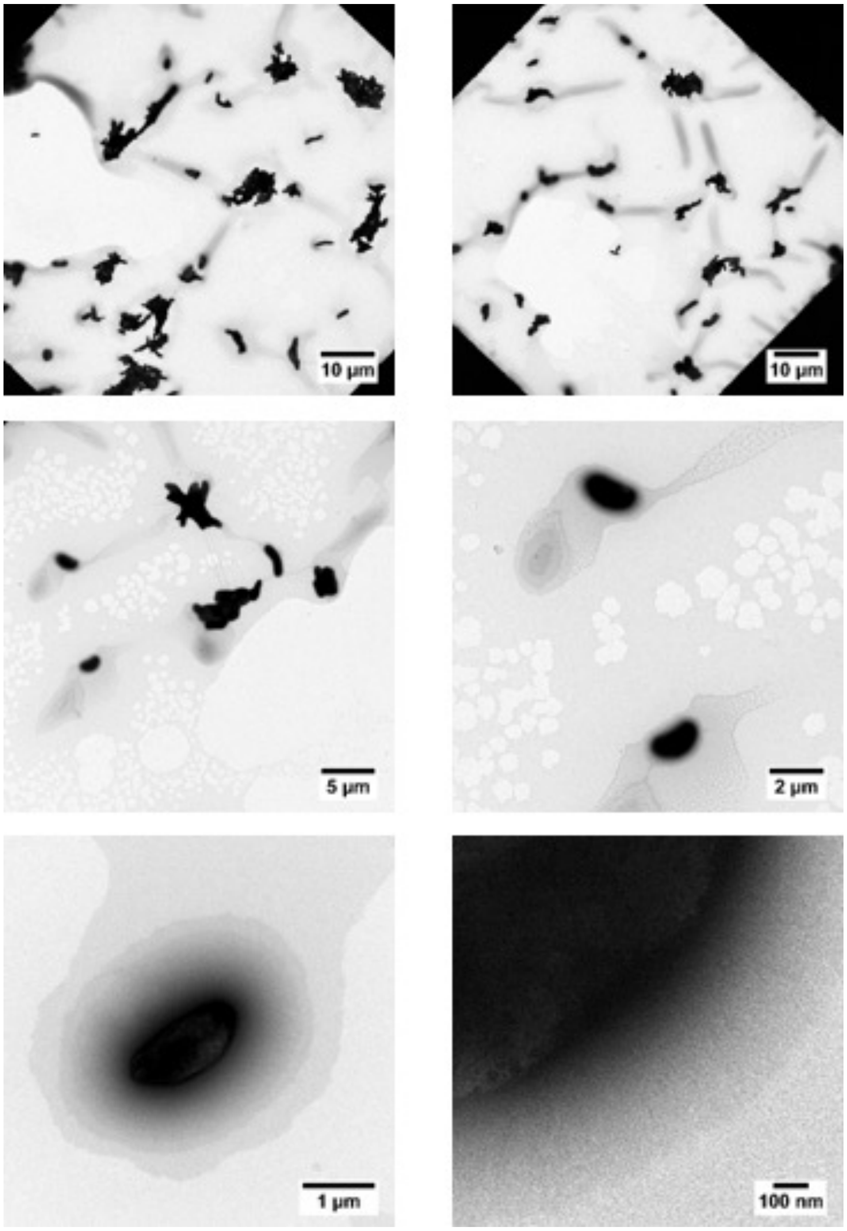

Fig. S7 *Negative stained DaPdNPs in the presence of 0.4% w/v TPGS-1000*

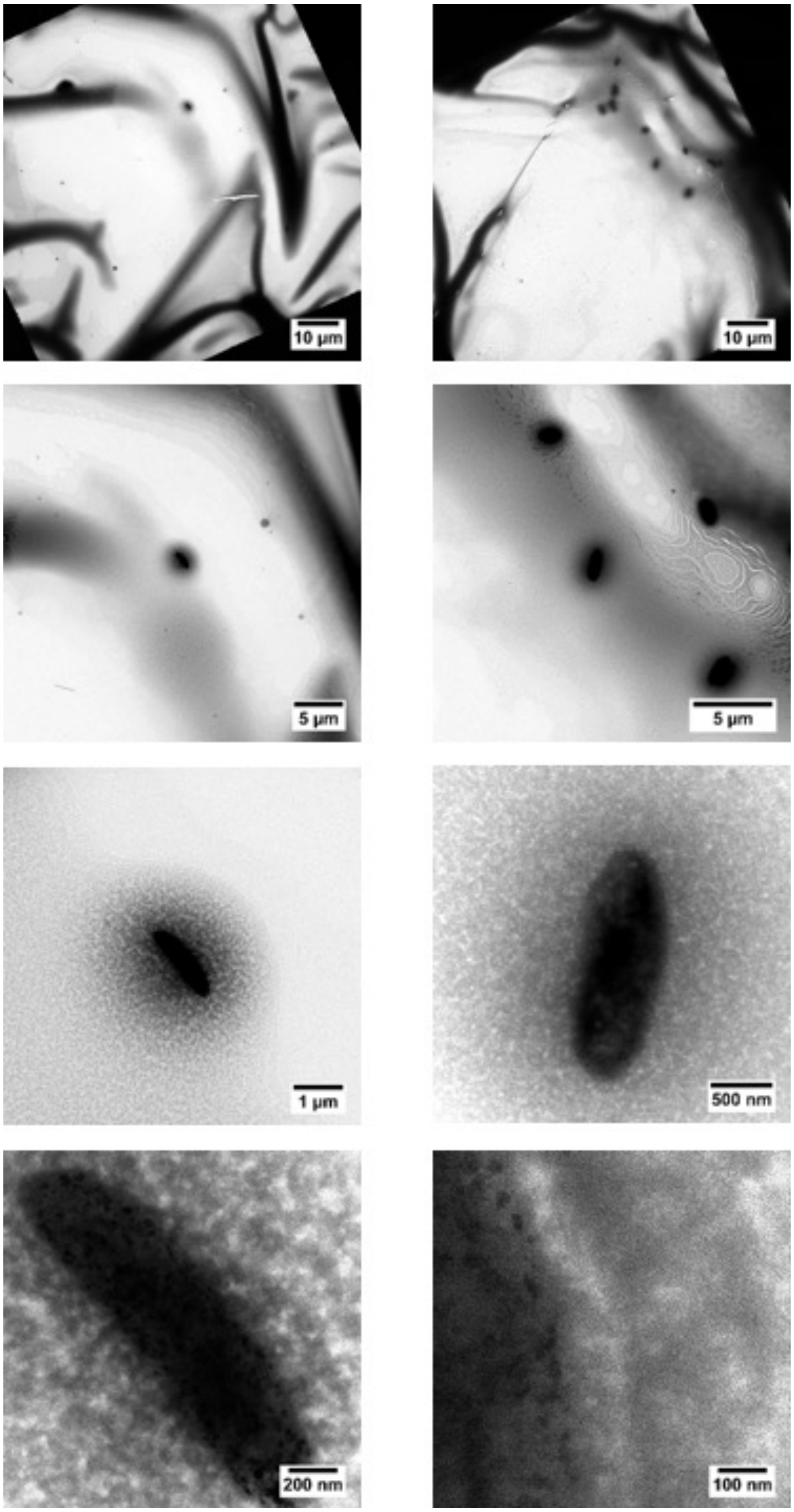

Fig. S8 *Negative stained DaPdNPs in the presence of 0.4% w/v TPGS-750-M*

S12 Product and Pd separation

The model reaction containing TPGS-1000 was conducted, extracted and concentrated as described above. The crude residue was dissolved in 10% ethanol (5 mL). An aliquot of the filtered solution (1 mL) was diluted in 1-methoxy-2-propanol to a final volume of 5 mL and analysed by ICP-OES immediately. As a result, no detectable amount of Pd was quantified in the sample (the calculated LoD[3]: 0.02 μM). This demonstrated the cross-coupling product can be easily separated from *Da*PdNPs catalyst in the extraction process, meaning no extra separation steps are required.

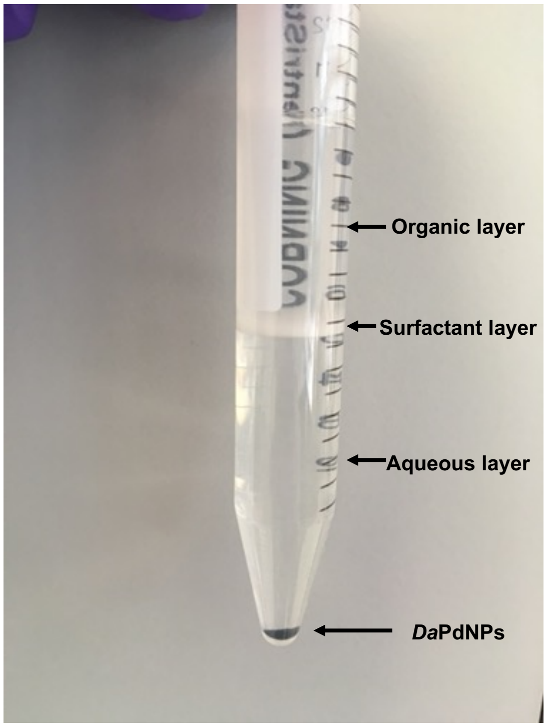

Fig. S9 *Pelleted DaPdNPs during liquid-liquid extraction*

S13 Product Characterisation

4-Methoxybiphenyl

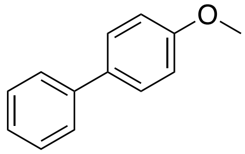

^{1}H NMR (500 MHz, $CDCl_3$): δ = 7.31–7.25 (m, 2H), 7.27–7.24 (m, 2H), 7.17–7.10 (m, 2H), 7.06–6.98 (m, 1H), 6.80–6.62 (m, 2H), 3.57 (s, 3H).
Spectroscopic data in good agreement with the literature.[4]

4'-Methyl-4-biphenylcarboxylate

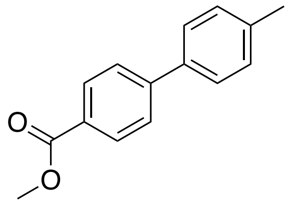

^{1}H NMR (500 MHz, DMSO): δ = 8.15–8.08 (m, 2H), 7.91–7.85 (m, 2H), 7.74–7.68 (m, 2H), 7.43–7.36 (m, 2H), 3.97 (s, 3H), 2.45 (s, 3H).
Spectroscopic data in good agreement with the literature.[5]

1-(4'-Methyl-4-biphenylyl)ethenone

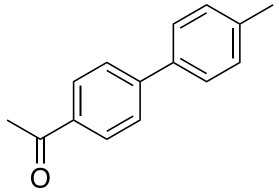

^{1}H NMR (500 MHz, $CDCl_3$): δ = 8.13–8.09 (m, 2H), 7.78–7.74 (m, 2H), 7.66–7.60 (m, 2H), 7.41–7.35 (m, 2H), 2.72 (s, 3H), 2.51 (s, 3H).
Spectroscopic data in good agreement with the literature.[5]

1-[4-(2-Methoxy-3-pyridinyl)phenyl]ethenone

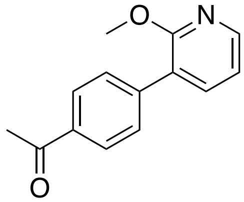

^{1}H NMR (500 MHz, $CDCl_3$): δ = 8.30 (dd, *J* = 4.9, 1.9, 1H), 8.17–8.05 (m, 2H), 7.78–7.75 (m, 2H), 7.74 (dd, *J* = 7.3, 1.9, 1H), 7.10 (dd, *J* = 7.3, 5.0, 1H), 4.09 (s, 3H), 2.74 (s, 3H).
Spectroscopic data in good agreement with the literature.[6]

1-(4'-Ethoxy-4-biphenylyl)ethenone

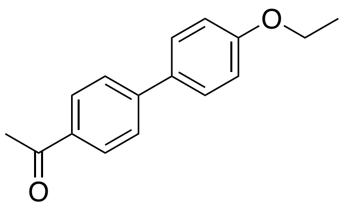

^{1}H NMR (500 MHz, $CDCl_3$): δ = 8.13–8.05 (m, 2H), 7.76–7.71 (m, 2H), 7.70–7.61 (m, 2H), 7.10–7.06 (m, 2H), 4.19 (q, *J* = 7.0, 2H), 2.72 (s, 3H), 1.55 (t, *J* = 7.0, 3H).
Spectroscopic data in good agreement with the literature.[7]

1-(3'-Ethoxy-4-biphenylyl)ethenone

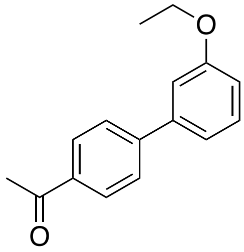

^{1}H NMR (500 MHz, $CDCl_3$): δ = 8.17–8.03 (m, 2H), 7.81–7.69 (m, 2H), 7.47 (t, *J* = 7.9, 1H), 7.29 (ddd, *J* = 7.6, 1.8, 0.9, 1H), 7.25 (dd, *J* = 2.5, 1.7, 1H), 7.03 (ddd, *J* = 8.3, 2.5, 0.9, 1H), 4.20 (q, *J* = 7.0, 2H), 2.73 (s, 3H), 1.55 (t, *J* = 7.0, 3H).

1-(2'-Ethoxy-4-biphenylyl)ethenone

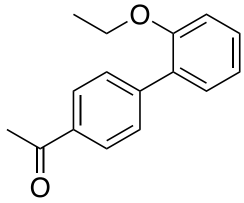

^{1}H NMR (500 MHz, $CDCl_3$): δ = 8.11–8.07 (m, 2H), 7.79–7.71 (m, 2H), 7.51–7.40 (m, 2H), 7.13 (td, *J* = 7.5, 1.1, 1H), 7.11–7.03 (m, 1H), 4.15 (q, *J* = 7.0, 2H), 2.73 (s, 3H), 1.45 (t, *J* = 7.0, 3H).

1-(4'-Methyl-3-biphenylyl)ethenone

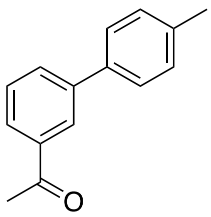

^{1}H NMR (500 MHz, $CDCl_3$): δ = 8.27 (t, *J* = 1.8, 1H), 8.01 (dt, *J* = 7.7, 1.5, 1H), 7.87 (ddd, *J* = 7.7, 1.9, 1.1, 1H), 7.64–7.59 (m, 3H), 7.37 (d, *J* = 7.8, 2H), 2.75 (s, 3H), 2.51 (s, 3H).
Spectroscopic data in good agreement with the literature.[5]

1-[3-(2-Methoxy-3-pyridinyl)phenyl]ethenone

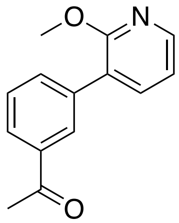

^{1}H NMR (500 MHz, $CDCl_3$): δ = 8.30 (dd, *J* = 5.0, 1.9, 1H), 8.24 (t, *J* = 1.7, 1H), 8.04 (ddd, *J* = 7.7, 1.9, 1.1, 1H), 7.87 (ddd, *J* = 7.7, 1.9, 1.2, 1H), 7.74 (dd, *J* = 7.3, 1.9, 1H), 7.62 (t, *J* = 7.7, 1H), 7.10 (dd, *J* = 7.3, 5.0, 1H), 4.09 (s, 3H), 2.74 (s, 3H).

1-(4'-Ethoxy-3-biphenylyl)ethenone

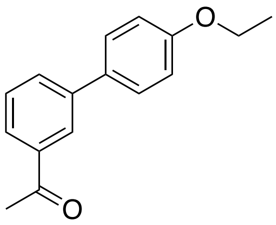

^{1}H NMR (500 MHz, $CDCl_3$): δ = 8.24 (t, *J* = 1.8, 1H), 7.98 (ddd, *J* = 7.7, 1.7, 1.1, 1H), 7.85 (ddd, *J* = 7.8, 1.9, 1.1, 1H), 7.68–7.63 (m, 2H), 7.60 (t, *J* = 7.7, 1H), 7.10–7.06 (m, 2H), 4.18 (q, *J* = 7.0, 2H), 2.75 (s, 3H), 1.55 (t, *J* = 7.0, 3H).

1-(3'-Ethoxy-3-biphenylyl)ethenone

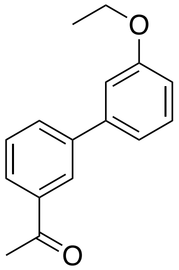

^{1}H NMR (500 MHz, $CDCl_3$): δ = 8.27 (t, *J* = 1.9, 1H), 8.03 (ddd, *J* = .7, 1.8, 1.2, 1H), 7.88 (ddd, *J* = 7.7, 1.9, 1.1, 1H), 7.63 (t, *J* = 7.7, 1H), 7.49–7.44 (m, 1H), 7.29 (ddd, *J* = 7.6, 1.7, 0.9, 1H), 7.24 (dd, *J* = 2.5, 1.7, 1H), 7.02 (ddd, *J* = 8.2, 2.5, 0.9, 1H), 4.21 (q, *J* = 7.0, 2H), 2.75 (s, 3H), 1.55 (t, *J* = 6.9, 3H).

1-(2'-Ethoxy-3-biphenylyl)ethenone

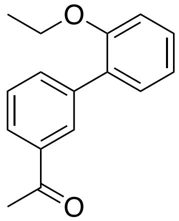

^{1}H NMR (500 MHz, $CDCl_3$): δ = 8.29 (t, *J* = 1.8, 1H), 8.02 (dt, *J* = 7.8, 1.5, 1H), 7.86 (dt, *J* = 7.6, 1.4, 1H), 7.59 (t, *J* = 7.7, 1H), 7.47–7.39 (m, 2H), 7.14 (td, *J* = 7.5, 1.1, 1H), 7.09 (dd, *J* = 8.2, 1.1, 1H), 4.16 (q, *J* = 7.0, 2H), 2.73 (s, 3H), 1.46 (t, *J* = 7.0, 3H).

1-(4'-Methyl-2-biphenylyl)ethenone

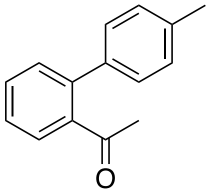

^{1}H NMR (500 MHz, $CDCl_3$): δ = 7.64 (dd, *J* = 7.7, 1.5, 1H), 7.63–7.54 (m, 1H), 7.52–7.46 (m, 2H), 7.33 (s, 4H), 2.51 (s, 3H), 2.47 (s, 3H).
Spectroscopic data in good agreement with the literature.[8]

1-(4'-Ethoxy-2-biphenylyl)ethenone

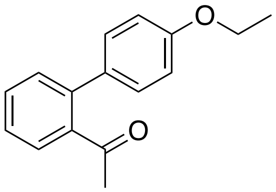

^{1}H NMR (500 MHz, $CDCl_3$): δ = 7.86–7.82 (m, 1H), 7.64–7.59 (m, 1H), 7.60–7.53 (m, 1H), 7.51–7.45 (m, 1H), 7.38–7.32 (m, 2H), 7.08–7.02 (m, 2H), 4.22 (q, *J* = 6.9, 2H), 2.11 (s, 3H), 1.56 (t, *J* = 6.9, 3H).

1-(3'-Ethoxy-2-biphenylyl)ethenone

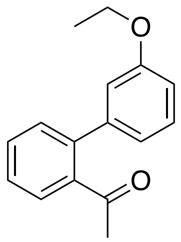

^{1}H NMR (500 MHz, $CDCl_3$): δ = 7.63 (dd, *J* = 7.4, 1.5, 1H), 7.60 (td, *J* = 7.5, 1.5, 1H), 7.52 (dd, *J* = 7.4, 1.2, 2H), 7.51–7.46 (m, 1H), 7.03 (ddd, *J* = 8.3, 2.5, 1.0, 1H), 7.01–6.99 (m, 1H), 6.98 (dd, *J* = 2.5, 1.6, 1H), 4.19 (d, *J* = 6.9, 2H), 2.13 (s, 3H), 1.53 (t, *J* = 7.1, 3H).

1-(2'-Ethoxy-2-biphenylyl)ethenone

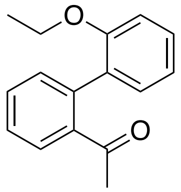

^{1}H NMR (500 MHz, $CDCl_3$): δ = 7.76–7.69 (m, 1H), 7.60 (td, *J* = 7.6, 1.5, 1H), 7.41 (s, 2H), 7.14 (dd, *J* = 7.5, 1.1, 1H), 7.04–7.00 (m, 3H), 4.07 (q, *J* = 7.0, 2H), 2.22 (s, 3H), 1.51 (t, *J* = 7.0, 3H).

4-Methyl-4'-nitrobiphenyl

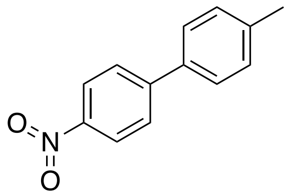

^{1}H NMR (500 MHz, $CDCl_3$): δ = 8.39–8.35 (m, 2H), 7.83–7.80 (m, 2H), 7.65–7.60 (m, 2H), 7.40 (d, J=7.9, 2H), 2.52 (s, 3H).
Spectroscopic data in good agreement with the literature.[9]

2-Methoxy-3-(4-nitrophenyl)pyridine

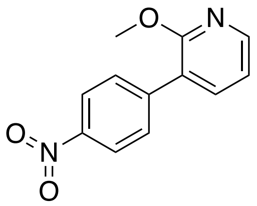

^{1}H NMR (500 MHz, $CDCl_3$) δ = 8.40–8.36 (m, 2H), 8.34 (dd, *J* =5.0, 1.8, 1H), 7.86–7.83 (m, 2H), 7.76 (dd, *J* = 7.3, 1.9, 1H), 7.13 (dd, *J* = 7.3, 5.0, 1H), 4.04 (s, 3H).

4-Ethoxy-4'-nitrobiphenyl

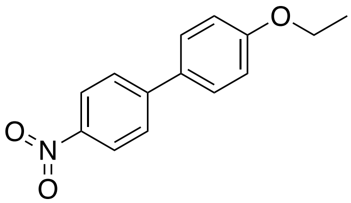

^{1}H NMR (500 MHz, $CDCl_3$): δ = 8.38–8.33 (m, 2H), 7.81–7.76 (m, 2H), 7.69–7.64 (m, 2H), 7.12–7.08 (m, 2H), 4.20 (q, *J* = 6.9, 2H), 1.55 (t, *J* = 7.0, 3H).
Spectroscopic data in good agreement with the literature.[7]

3-Ethoxy-4'-nitrobiphenyl

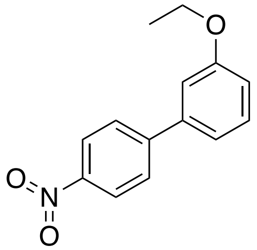

^{1}H NMR (500 MHz, $CDCl_3$): δ = 8.40–8.36 (m, 2H), 7.84–7.80 (m, 2H), 7.49 (t, *J* = 7.9, 1H), 7.29 (ddd, *J* = 7.7, 1.8, 0.9, 1H), 7.24 (dd, *J* = 2.5, 1.7, 1H), 7.07 (ddd, *J* = 8.2, 2.5, 0.9, 1H), 4.21 (q, *J* = 7.0, 2H), 1.56 (t, *J* = 7.0, 3H).

2-Ethoxy-4'-nitrobiphenyl

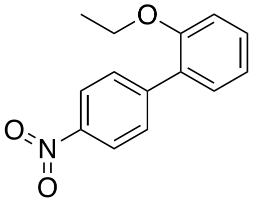

^{1}H NMR (500 MHz, $CDCl_3$): δ = 8.37–8.32 (m, 2H), 7.84–7.80 (m, 2H), 7.47 (ddd, J = 8.2, 7.4, 1.8, 1H), 7.44 (dd, J = 7.6, 1.7, 1H), 7.15 (td, J = 7.5, 1.1, 1H), 7.10 (dd, J = 8.3, 1.0, 1H), 4.17 (q, J = 7.0, 2H), 1.46 (t, J = 7.0, 3H).

4'-Methyl-4-biphenylol

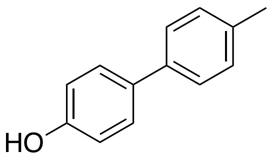

^{1}H NMR (500 MHz, $CDCl_3$): δ = 7.54 (dd, J = 4.5, 2.0, 2H), 7.53–7.51 (m, 2H), 7.32–7.29 (m, 2H), 7.00–6.96 (m, 2H), 2.47 (s, 3H).
Spectroscopic data in good agreement with the literature.[10]

2-Methoxy-3-(4-phenylol)pyridine

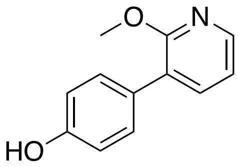

^{1}H NMR (500 MHz, $CDCl_3$): δ = 8.21 (dd, J = 5.0, 1.9, 1H), 7.69 (dd, J = 2.0, 0.9, 1H), 7.55–7.50 (m, 2H), 7.04 (dd, J = 7.2, 5.0, 1H), 7.01–6.98 (m, 2H), 3.87 (s, 3H).

4'-Ethoxy-4-biphenylol

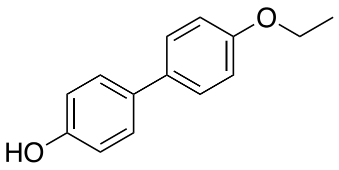

^{1}H NMR (500 MHz, $CDCl_3$): δ = 7.56–7.52 (m, 2H), 7.51–7.48 (m, 2H), 7.05–7.01 (m, 2H), 6.99–6.95 (m, 2H), 4.15 (q, J = 7.1, 2H), 1.52 (t, J = 7.0, 3H).

3'-Ethoxy-4-biphenylol

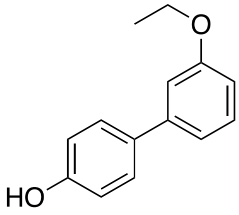

^{1}H NMR (500 MHz, $CDCl_3$): δ = 7.57–7.52 (m, 2H), 7.39 (t, *J* = 7.9, 1H), 7.21 (ddd, *J* = 7.7, 1.7, 1.0, 1H), 7.17 (dd, *J* = 2.5, 1.7, 1H), 7.01–6.97 (m, 2H), 6.92 (ddd, *J* = 8.2, 2.6, 0.9, 1H), 4.18 (q, *J* = 7.0, 2H), 1.53 (t, *J* = 7.0, 3H).

2'-Ethoxy-4-biphenylol

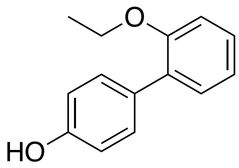

^{1}H NMR (500 MHz, $CDCl_3$): δ = 7.41–7.35 (m, 3H), 7.01 (dt, *J* = 7.4, 1.1, 1H), 6.99 (dt, *J* = 7.8, 1.0, 2H), 6.83–6.78 (m, 2H), 4.13 (q, *J* = 7.0, 2H), 1.51 (t, *J* = 7.0, 3H).

All NMR spectra are available: https://doi.org/10.7488/ds/3108

S14 References

[1] M. J. Capeness, V. Echavarri-Bravo, L. E. Horsfall, *Front. Microbiol.* 2019, **10**, 997.

[2] S. S. Wang, *J. Am. Chem. Soc.* 1973, **95**,1328.

[3] D. A. Armbruster, T. Pry, *Clin. Biochem. Rev*. 2008, **29**, S49.

[4] E. Alacid, C. Nájera, *Org. Lett.* 2008, **10**, 5011.

[5] M. Tobisu, T. Xu, T. Shimasaki, N. Chatani, *J. Am. Chem. Soc.* 2011, **133**, 19505.

[6] M. A. Pena, J. P. Sestelo, L. A. Sarandeses, *J. Org. Chem.* 2007, **72**, 1271.

[7] P. Lan, D. Berta, J. A. Porco, M. S. South, J. J. Parlow, *J. Org. Chem.* 2003, **68**, 9678.

[8] L. J. Goossen, N. Rodríguez, C. Linder, *J. Am. Chem. Soc.* 2008, **130**, 15248.

[9] M. L. N. Rao, D. N. Jadhav, D. Banerjee, *Tetrahedron* 2008, **64**, 5762.

[10] S. A. Moteki, J. M. Takacs, *Angew.Chem. Int. Ed.* 2008, **47**, 894.
